# An Input-Current Shaping and Soft-Switching Drive Circuit Applied to a Piezoelectric Ceramic Actuator

**DOI:** 10.3390/mi14101906

**Published:** 2023-10-05

**Authors:** Chun-An Cheng, Hung-Liang Cheng, Chien-Hsuan Chang, En-Chih Chang, Long-Fu Lan, Hao-Fang Hsu

**Affiliations:** Department of Electrical Engineering, I-Shou University, Kaohsiung 84001, Taiwan; cacheng@isu.edu.tw (C.-A.C.); hlcheng@isu.edu.tw (H.-L.C.); chchang@isu.edu.tw (C.-H.C.); isu11001004m@cloud.isu.edu.tw (L.-F.L.); isu10901010m@cloud.isu.edu.tw (H.-F.H.)

**Keywords:** drive circuit, input-current shaping, piezoelectric ceramic actuator, soft-switching, zero-voltage switching

## Abstract

Piezoelectric ceramic actuators utilize an inverse piezoelectric effect to generate high-frequency vibration energy and are widely used in ultrasonic energy conversion circuits. This paper presents a novel drive circuit with input-current shaping (ICS) and soft-switching features which consists of a front AC-DC full-wave bridge rectifier and a rear DC-AC circuit combining a stacked boost converter and a half-bridge resonant inverter for driving a piezoelectric ceramic actuator. To enable ICS functionality in the proposed drive circuit, the inductor of the stacked boost converter sub-circuit is designed to operate in boundary-conduction mode (BCM). In order to allow the two power switches in the proposed drive circuit to achieve zero-voltage switching (ZVS) characteristics, the resonant circuit of the half-bridge resonant inverter sub-circuit is designed as an inductive load. In this paper, a prototype drive circuit for providing piezoelectric ceramic actuators was successfully implemented. Experimental results tested at 110 V input utility voltage show that high power factor (PF > 0.97), low input current total harmonic distortion (THD < 16%), and ZVS characteristics of the power switch were achieved in the prototype drive circuit.

## 1. Introduction

In 1880, two French physicists, Pierre Curie and Jacques Curie, were investigating the relationship between the phenomenon of thermo-electricity and crystals. They found that when an external force was applied to a crystal, electric polarization occurred inside the crystal. Subsequently, when they applied an electric field to the crystal, this caused a deformation on the outside of the crystal, a phenomenon known as the piezoelectric effect. The piezoelectric effect is a phenomenon in which mechanical energy and electrical energy are mutually exchanged in dielectric materials. The piezoelectric effect is used in the generation and detection of sound, the generation of high voltages, the generation of electric frequencies, microbalances and the ultra-fine focusing of optical devices. There are two types of piezoelectric effect: positive piezoelectric effect and inverse piezoelectric effect. When physical pressure is applied to the piezoelectric material, the electric dipole moment in the material body will be shortened due to compression. At this time, the piezoelectric material will generate equal positive and negative charges on the opposite surfaces of the material to maintain the original state in order to resist this change. This phenomenon of electrical polarization due to deformation is called the “positive piezoelectric effect”. When an electric field is applied on the surface of the piezoelectric material, the electric dipole moment will be elongated due to the action of the electric field, and the piezoelectric material will elongate along the direction of the electric field in order to resist the change. This process of generating mechanical deformation through the action of an electric field is called the “inverse piezoelectric effect” [1,2,3,4,5,6].

Piezoelectric materials have a piezoelectric effect because of the special arrangement of atoms in the crystal lattice, which makes the material have the effect of stress field and electric field coupling. And piezoelectric ceramics are a kind of piezoelectric materials belonging to piezoelectric polycrystalline materials. The main function of piezoelectric ceramics is to convert mechanical and electrical energy into each other. When pressure is applied to a piezoelectric ceramic, a potential difference is generated. When a voltage is applied to a piezoelectric ceramic, a mechanical stress is generated. If a high-frequency vibration is applied to the piezoelectric ceramic, it will produce a high-frequency current. If a high-frequency electrical signal is applied to the piezoelectric ceramic, it will generate a high-frequency mechanical vibration. One application of piezoelectric ceramics is to use them as an actuator. A piezoelectric ceramic actuator is an electromechanical device that utilizes the piezoelectric effect to convert electrical energy into mechanical motion or displacement. The piezoelectric effect is a property exhibited by certain materials (especially piezoelectric ceramics) which generate an electric charge when they are subjected to mechanical stress and, conversely, deform when they are subjected to an electric field. Since piezoelectric ceramics deform and vibrate under the action of an electric field, resonance occurs when the frequency of the electric field is the same as one of the intrinsic frequencies of the piezoelectric ceramics. Since the amplitude of piezoelectric ceramics at resonance is hundreds of times greater than at normal frequencies, piezoelectric ceramics can be used as an actuator to convert into mechanical energy with maximum efficiency [7,8].

In a piezoelectric ceramic actuator, a piezoelectric ceramic material is typically sandwiched between electrodes. When a voltage is applied to an electrode, the piezoelectric material deforms, causing it to expand or contract. This deformation produces mechanical motion or displacement and can be used in a variety of applications. Piezoelectric ceramic actuators are known for their high precision, fast response time, and large force-to-size ratios, and are used in a wide range of applications, including: (1) Applications of precision positioning—piezoelectric ceramic actuators are used in fine positioning stages and nanometer-scale positioning systems, such as those found in microscopy, semiconductor manufacturing, and optical equipment. (2) Applications of valve and pump actuation—piezoelectric ceramic actuators can control valves and pumps in fluidic systems with high accuracy and rapid response times. (3) Applications of ultrasound and medical imaging—piezoelectric ceramic actuators are used in medical imaging devices like ultrasound transducers, where they emit and receive ultrasonic waves. (4) Applications in vibration and noise control—piezoelectric ceramic actuators are employed in active vibration damping and noise cancellation systems in various industries, including aerospace and automotive. (5) Applications of inkjet printing—piezoelectric ceramic actuators are used to control droplet ejection in inkjet printers. (6) Applications of precision machining—piezoelectric ceramic actuators are used in machining applications that require high precision and stability, such as in laser micromachining. (7) Applications of aerospace and defense—piezoelectric ceramic actuators are used in aerospace applications for mechanisms such as adaptive structures and morphing wing technology. (8) Applications in haptic feedback—piezoelectric ceramic actuators provide tactile feedback in electronic devices, such as touchscreens and gaming controllers. (9) Applications of energy harvesting—piezoelectric ceramic actuators can also be used to convert mechanical vibrations into electrical energy, a process known as energy harvesting [9,10,11,12,13,14,15,16,17].

Since piezoelectric ceramic actuators uses the inverse piezoelectric effect to convert electrical energy into mechanical energy and generate high-frequency vibration energy, they can be widely used in low-power ultrasonic energy conversion circuits such as ultrasonic beauty instruments and tooth cleaners, as well as in ultrasonic cleaning machines, ultrasonic processing machines, and other high-power ultrasonic energy conversion circuits [18,19,20,21,22].

**Figure 1:**
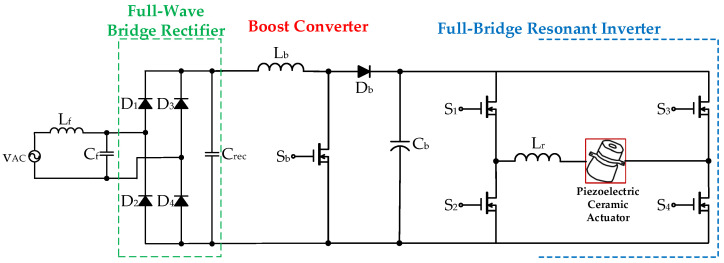
Three-stage drive circuit suitable for supplying a piezoelectric ceramic actuator applied with an AC input voltage source with ICS function.

The general three-stage piezoelectric ceramic drive circuit is composed of a full-wave bridge rectifier in the front stage, a DC-DC converter with input-current shaping (ICS) function in the middle stage, and a DC-AC resonant converter in the rear stage. Reference [23] has presented a drive circuit for powering a piezoelectric ceramic actuator applied with a DC input voltage source, and it can be extended to a three-stage driver circuit using an AC input voltage source. Figure 1 shows the three-stage drive circuit for powering a piezoelectric ceramic actuator using an AC input voltage source *v_AC_* and with ICS function, which is composed of a front-stage AC-DC full-wave bridge rectifier (including four diodes, *D*_1_, *D*_2_, *D*_3_, and *D*_4_ along with a capacitor, *C_rec_*), a middle-stage DC-DC boost converter (including a capacitor, *C_rec_*, an inductor, *L_b_*, a power switch, *S_b_*, a diode, *D_b_* and a DC-linked capacitor, *C_b_*), and a rear-stage DC-AC full-bridge resonant converter (including a DC-linked capacitor, *C_b_*, four power switches, *S*_1_, *S*_2_, *S*_3_, *S*_4_, and a resonant inductor, *L_r_*) that supply the piezoelectric ceramic actuator with rated power.

**Figure 2:**
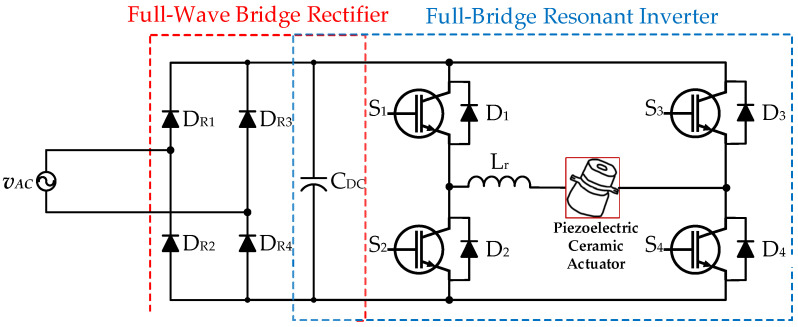
The conventional two-stage drive circuit for supplying a piezoelectric ceramic actuator applied with an AC input voltage source without PFC [24,25].

The conventional two-stage drive circuit for providing a piezoelectric ceramic actuator applied with an AC input voltage source *v*_AC_ is shown in Figure 2 [24,25], and consists of a front-stage AC-DC full-wave bridge rectifier (including four diodes, *D*_R1_, *D*_R2_, *D*_R3_, and *D*_R4_, along with a DC-linked capacitor, *C_DC_*) and a rear-stage DC-AC full-bridge resonant converter (including four power switches, *S*_1_, *S*_2_, *S*_3_, and *S*_4_, four diodes, *D*_1_, *D*_2_, *D*_3_, and *D*_4_, and a resonant inductor, *L*_r_) that supplies the piezoelectric ceramic actuator with rated power. In addition, this two-stage version, which powers piezo-ceramic actuators, does not have a power factor correction (PFC) function.

The above-mentioned three-stage drive circuit supplies power to the piezoelectric ceramic driver and uses an AC input voltage source with ICS function, which requires three-level power conversion. In addition, the number of power switches in the three-stage conversion circuit is relatively large, so the switching loss and conduction loss are relatively large, which affects the overall efficiency of the circuit. Typically, electrical equipment powered by an AC source requires power factor correction, which is important and necessary to increase the power factor to as close to unity as possible to save energy, reduce greenhouse gas emissions, and reduce the consumption of fossil fuels at power stations in order to improve energy efficiency. The two-stage drive circuit described above uses an AC input voltage source to power the piezoelectric ceramic driver, but does not provide power factor correction. In response to these challenges, this paper proposes a novel two-stage drive circuit to provide piezoelectric ceramic actuators with ICS and soft-switching functions, which consists of a full-wave bridge rectifier in the front stage and a stacked boost-half-bridge resonant converter in the rear stage. By designing the series inductance of the stacked boost-half-bridge resonant converter to operate in the boundary-conduction mode (BCM), the drive circuit has ICS function. In addition, the resonant tank circuit of the stacked boost-half bridge resonant converter is designed to operate as an inductive load. Thus, two power switches of the driver circuit can realize zero-voltage switching (ZVS) characteristics [26]. This paper, which is an extended version of [26], is organized as follows. Section 2 not only describes and analyzes operational modes in the proposed two-stage drive circuit for supplying a piezoelectric ceramic actuator, but also presents a design guideline of the circuit parameter in the proposed drive circuit. In Section 3, experimental results of the prototype drive circuit for supplying a piezoelectric ceramic actuator are demonstrated. Finally, some conclusions are provided in Section 4.

## 2. The Proposed Two-Stage Drive Circuit for Supplying a Piezoelectric Ceramic Actuator

### 2.1. Introduction of the Proposed Two-Stage Drive Circuit

**Figure 3:**
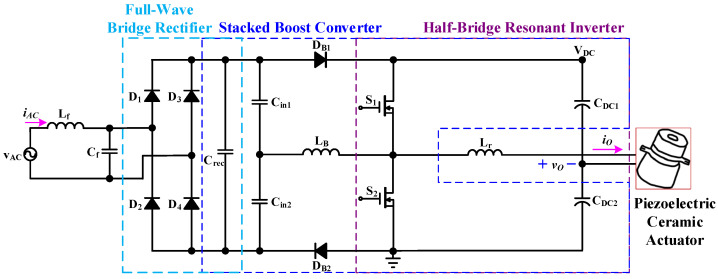
The proposed two-stage driver circuit for providing a piezoelectric ceramic actuator.

Figure 3 shows the proposed drive circuit for providing a piezoelectric ceramic actuator, which combines a front-stage AC-DC full-wave bridge rectifier and a rear-stage circuit that integrates a stacked boost converter with a half-bridge resonant inverter. The stacked boost converter sub-circuit is composed of two capacitors (*C*_in1_ and *C*_in2_), two diodes (*D_B_*_1_ and *D_B_*_2_), an inductor *L_B_*, two power switches (*S*_1_ and *S*_2_), and two DC-linked capacitors (*C_DC_*_1_ and *C_DC_*_2_). The half-bridge resonant inverter sub-circuit is composed of two switches (*S*_1_ and *S*_2_), two DC-linked capacitors (*C_DC_*_1_ and *C_DC_*_2_), a resonant inductor (*L_r_*), and the piezoelectric ceramic actuator. Moreover, the inductor *L_B_* is designed to be operated in BCM in order to accomplish ICS function. And the resonant tank circuit design of the stacked dual boost-half-bridge resonant converter is operated under the inductive load, so that the two power switches can achieve the characteristics of zero-voltage switching (ZVS), thereby improving the conversion efficiency of the circuit and reducing the switching loss on the power switch.

### 2.2. Analysis of Operational Modes

**Figure 4:**
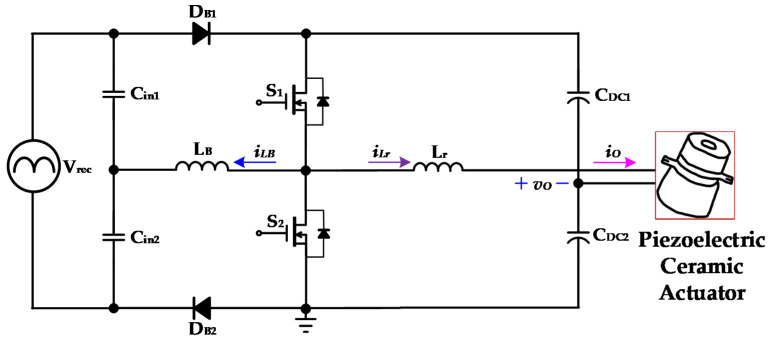
The equivalent driver circuit for providing a piezoelectric ceramic actuator during analysis of the operational modes.

Figure 4 is a simplified diagram for analyzing the operational mode of the presented drive circuit for powering the piezoelectric ceramic actuator. In analyzing the mode of operation of the novel drive circuit proposed in this paper for application to piezoelectric ceramic actuators, the following assumptions are made about some of the circuit components:

- The voltage source *Vrec* is defined as the voltage output from the input AC voltage to the capacitors *C_in_*_1_ and *C_in_*_2_ through the full-wave bridge rectifier of the previous stage.
- The gate-driving control signals of the power switches *S*_1_ and *S*_2_ are complementary, and the intrinsic diodes of the power switches are considered. The dead time of the two power switches *S*_1_ and *S*_2_ is ignored, and the duty ratio of the two power switches is assumed to be 0.5.
- The series inductor *L_B_* is designed to operate in boundary-conduction mode (BCM).
- When analyzing how the circuit works, the flow direction of the series inductor current is defined as the right-to-left direction, and the flow direction of the resonant inductor current is defined as the left-to-right direction.
- During the circuit analysis, ignore the equivalent resistance of diodes *D_B_*_1_ and *D_B_*_2_ and their forward bias voltage drop.
- The rest of the circuit elements can be considered as ideal elements.

**Figure 5:**
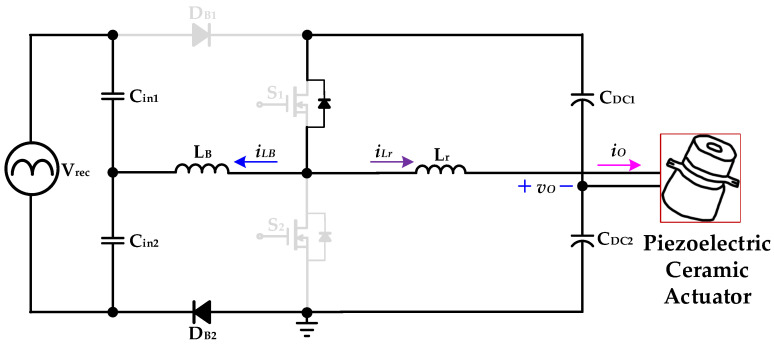
Operational mode 1 of the proposed drive circuit for the piezoelectric ceramic actuator.

**Figure 6:**
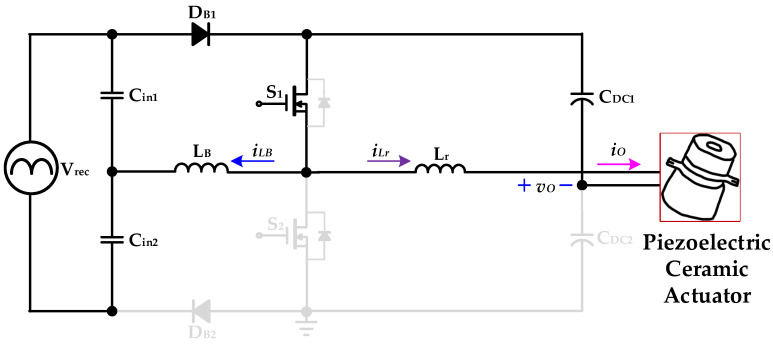
Operational mode 2 of the proposed drive circuit for the piezoelectric ceramic actuator.

**Figure 7:**
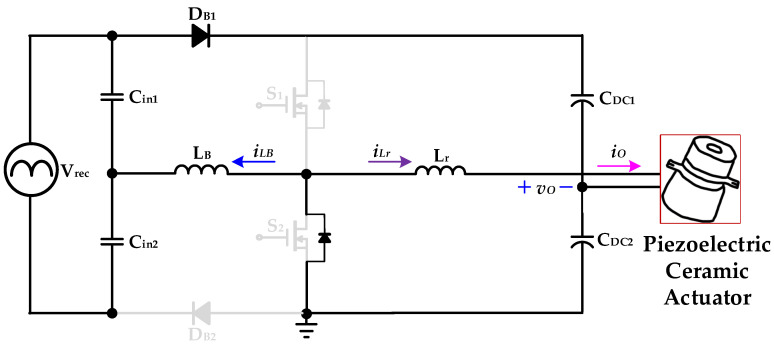
Operational mode 3 of the proposed drive circuit for the piezoelectric ceramic actuator.

**Figure 8:**
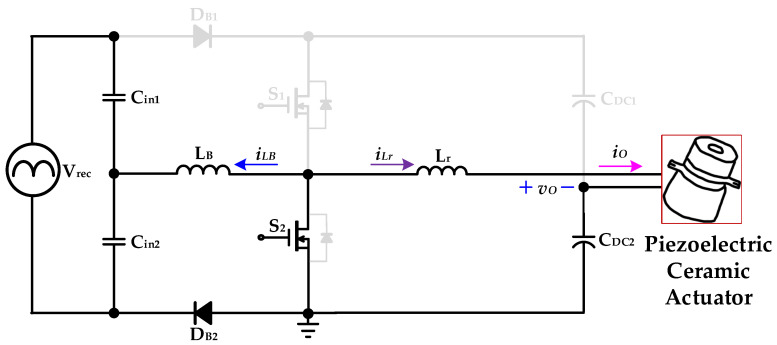
Operational mode 4 of the proposed drive circuit for the piezoelectric ceramic actuator.

**Figure 9:**
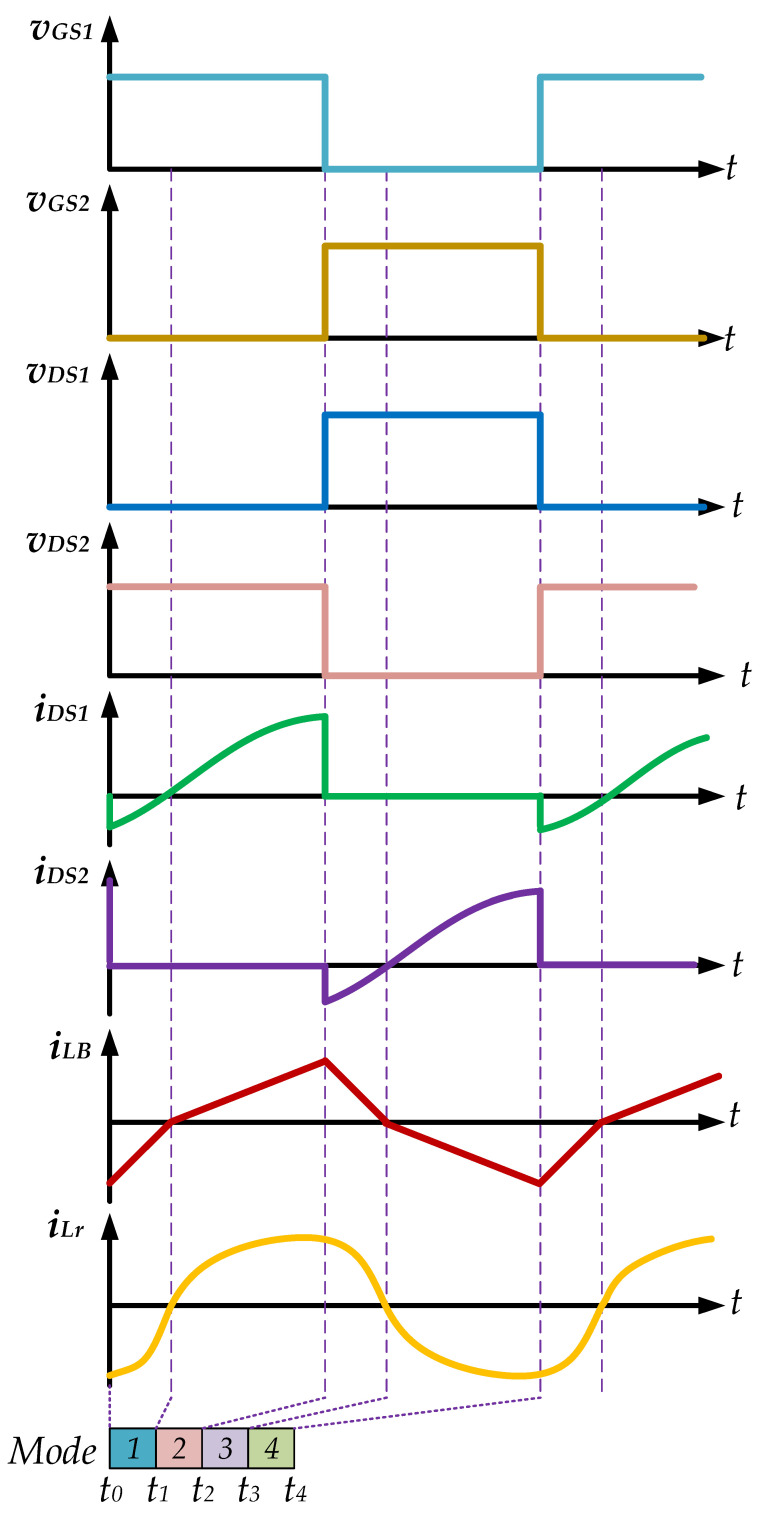
Theoretical waveforms of the proposed drive circuit of the piezoelectric ceramic actuator during the positive half-cycle of the utility-line voltage.

Figure 5, Figure 6, Figure 7 and Figure 8 are the analysis diagrams of operational mode 1 to operational mode 4 of the proposed driver circuit. Among them, operational mode 2 and operational mode 3 are the positive half cycle of the series inductor current *i_LB_*, and operational mode 1 and operational mode 4 are the negative half cycle of the series inductor current *i_LB_*. Figure 9 is a schematic diagram of the voltage and current waveforms on the essential components of the new drive circuit applied to piezoelectric ceramic actuators in each action mode.

#### 2.2.1. Operational Mode 1 (*t*_0_ ≤ *t* < *t*_1_)

Figure 5 shows operational mode 1 of the proposed drive circuit applied to the piezoelectric ceramic actuator. In the previous operational mode, the capacitor *C_in_*_2_ completed the provision of energy to the series inductor *L_B_* through the switch *S*_2_ and the diode *D_B_*_2_, and then the intrinsic diode of the power switch *S*_1_ was forward-biased at *t*_0_. The series inductor *L_B_* and the capacitor *C_in_*_2_ provide energy to the DC-linked capacitors *C_DC_*_1_ and *C_DC_*_2_ through the intrinsic diode of the power switch *S*_1_ and the diode *D_B_*_2_. The current of the series inductor *L_B_* decreases linearly during its negative half-cycle operation.

The resonant inductor *L_r_* provides energy to the DC-linked capacitor *C_DC1_* and the piezoelectric ceramic actuator via the intrinsic diode of the power switch *S*_1_. When the power switch *S*_1_ is driven and has the ZVS feature, and the current of the series inductance *L_B_* drops to zero, operational mode 1 ends at *t*_1_.

#### 2.2.2. Operational Mode 2 (*t*_1_ ≤ *t* < *t*_2_)

Figure 6 shows operational mode 2 of the proposed drive circuit applied to piezoelectric ceramic actuators. In the previous operation mode, the power switch *S*_1_ was ZVS turn-on at *t*_1_. The capacitor *C_in_*_1_ supplies energy to the series inductor *L_B_* through the diode *D_B_*_1_ and the power switch *S*_1_, and the series inductor current *i_LB_* rises linearly at its positive half cycle. The DC-linked capacitor *C_DC_*_1_ provides energy to the resonant inductor *L_r_* and the piezoelectric ceramic actuator through the power switch *S*_1_. When the power switch *S*_1_ is turned off and the current of the series inductor *L_B_* rises to the maximum value at its positive half cycle, operational mode 2 ends at *t*_2_.

#### 2.2.3. Operational Mode 3 (*t*_2_ ≤ *t* < *t*_3_)

Figure 7 shows operational mode 3 of the proposed drive circuit applied to piezoelectric ceramic actuators. In the previous operation mode, the energy storage in the series inductor *L_B_* was completed, and the intrinsic diode of the power switch *S*_2_ was forward-biased at *t*_2_. The series inductor *L_B_* and the capacitor *C_in_*_1_ provide energy to the DC-linked capacitors *C_DC_*_1_ and *C_DC_*_2_ through the intrinsic diode of the power switch *S*_2_ and diode *D_B_*_1_. Thus, the series inductor current *i_LB_* decreases linearly at its positive half-cycle.

In addition, the resonant inductor *L_r_* provides energy to the DC-linked capacitor *C_DC_*_2_ and the piezoelectric ceramic actuator through the intrinsic diode of the power switch *S*_2_. When the series inductor current *i_LB_* drops to zero at its positive half-cycle and the power switch *S*_2_ is driven and has ZVS, operational mode 3 ends at *t*_3_.

#### 2.2.4. Operational Mode 4 (*t_3_* ≤ *t* < *t_4_*)

Figure 8 shows operational mode 4 of the proposed drive circuit applied to the piezoelectric ceramic actuator. In the previous operation mode, the power switch *S*_2_ was ZVS turn-on at *t*_3_. The capacitor *C_in_*_2_ supplies energy to the series inductor *L_B_* through the diode *D_B_*_2_ and the power switch *S*_2_, and the series inductor current *i_LB_* rises linearly at its negative half-cycle.

The DC-linked capacitor *C_DC_*_2_ provides energy to the resonant inductor *L_r_* and the piezoelectric ceramic actuator through the power switch *S*_2_. When the power switch *S*_2_ turns off and the series inductor current *i_LB_* rises to the maximum value at its negative half-cycle, operational mode 4 ends at *t*_4_ and the circuit operation returns to mode 1.

### 2.3. Design Guideline of the Series Inductor L_B_

**Figure 10:**
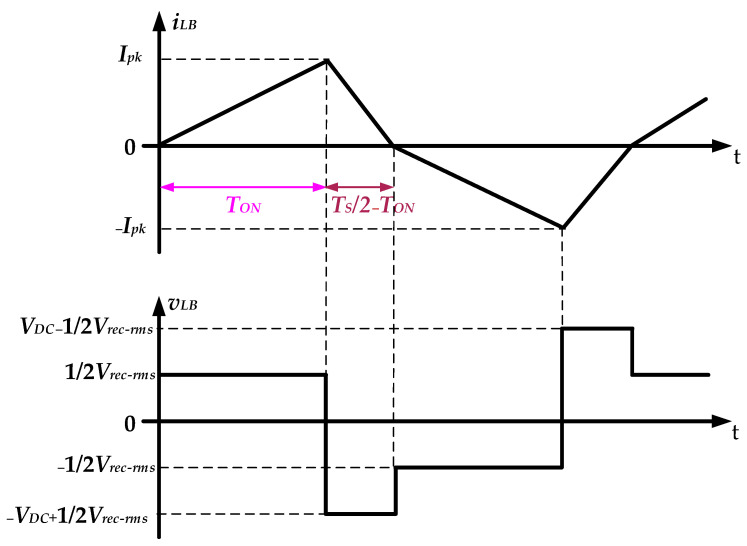
The theoretical current and voltage waveform *i_LB_* and *v_LB_* of the series inductor *L_B_*.

Figure 10 shows the theoretical current and voltage waveform *i_LB_* and *v_LB_* of the series inductor *L_B_*. By using the volt-second theorem, the peak value of the inductor current, denoted as *I_LB-peak_*, can be expressed by

(1) $$I_{LB-peak}=\frac{V_{rec-rms}}{2L_{B}}T_{ON}=\frac{V_{DC}-\frac{1}{2}V_{rec-rms}}{L_{B}}\left(\frac{1}{2}T_{S}-T_{ON}\right)$$

where *T_ON_* and *T_S_* are the turn-on time and the switching period of the power switches, respectively. From Equation (1), the turn-on time *T_ON_* of the power switch is given by

(2) $$T_{ON}=\frac{1}{2}-\frac{V_{rec-rms}}{4V_{DC}}$$

By combining (1) with (2), the design formula of the series inductor *L_B_* can be expressed as

(3) $$L_{B}=\frac{V_{rec-rms}}{2I_{LB-peak}}\times \left(\frac{1}{2}-\frac{V_{rec-rms}}{4V_{DC}}\right)T_{S}$$

With a *V_rec-rms_* of 110 V, a *V_DC_* of 400 V, a *I_LB-peak_* of 2 A, and a switching period *T_S_* of 1/(40 kHz), the inductances of the series inductor *L_B_* is calculated as

(4) $$L_{B}=\frac{110}{2\times 2}\times \left(\frac{1}{2}-\frac{110}{4\times 400}\right)\left(\frac{1}{40k}\right)=296.4\mu H$$

In addition, the series inductor *L*_B_ in the prototype drive circuit is 250 μH.

### 2.4. Design Equation of the Resonant Inductor L_r_

In order to achieve zero-voltage switching on both power switches and thus reduce switching losses, a resonant tank combining the series inductor *L_r_* with the equivalent circuit of the piezoelectric ceramic actuator is expected to be designed as an inductive load when the resonant frequency *f_r_* is equal to the switching frequency *f_s_*. Therefore, the design equation of the resonant inductor *L_r_* can be obtained as follows [25,27]:

(5) $$L_{r}=\frac{1}{2\pi f_{r}}\times \left(X_{1}+\sqrt{{\left|Z_{in}\right|}^{2}-R_{1}{}^{2}}\right)$$

where *Z_in_*, *R*_1_, and *X*_1_ are the input impedance, the equivalent resistance, and the reactance in the equivalent circuit of the piezoelectric ceramic actuator.

## 3. Experimental Results of the Proposed Drive Circuit

In this paper, a prototype of the proposed drive circuit for supplying a 50 W-rated piezoelectric ceramic actuator has already been implemented and testified. The specifications of the experimental piezoelectric ceramic actuator are shown in Table 1. The resonant resistance *R_m_*, the static capacitance *C_p_*, and the resonant frequency *f_r_* are 25 Ω, 4000 pF, and 40 ± 0.5 kHz, respectively. In addition, Table 2 lists the components used in the drive circuit of the piezoelectric ceramic actuator prototype.

**Figure 11:**
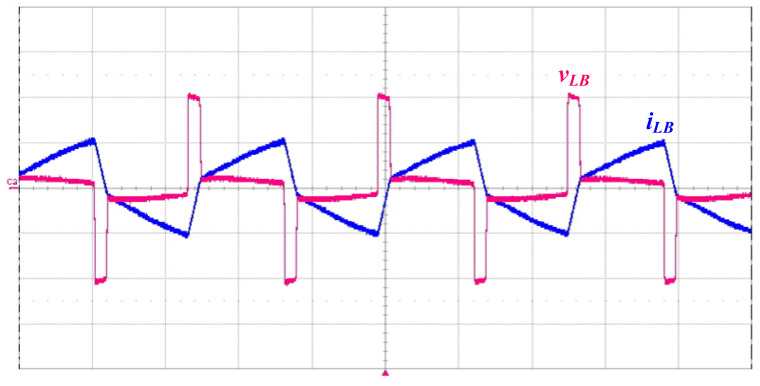
Measured waveform of the inductor voltage (200 V/div) and current *i_LB_* (2 A/div); time scale: 10 μs/div.

**Figure 12:**
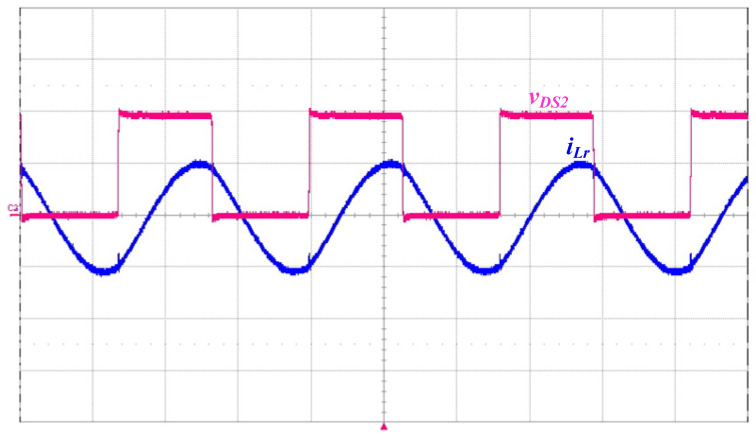
Measured waveforms of the switch voltage *v_DS_*_2_ (200 V/div) and the resonant inductor current *i_Lr_* (1 A/div); time scale: 10 μs/div.

In this paper, a prototype driver circuit for powering a piezoelectric ceramic actuator with a rated power of 50 W and a resonant frequency of 40 kHz has been successfully implemented and tested. Table 2 shows the circuit components used in the prototype piezoelectric ceramic actuator driver circuit. Figure 11 shows the measured inductor voltage *v_LB_* and current *i_LB_*, and the current *i_LB_* works in BCM, as can be seen from the figure. Figure 12 shows the measured waveforms of the switch voltage *v_DS_*_2_ and the resonant inductor current *i_Lr_*. It can be seen from the waveform diagram that the inductor current *i_Lr_* lags behind the switch voltage *v_DS_*_2_, so the series resonant circuit can be approximated as an inductive load.

**Figure 13:**
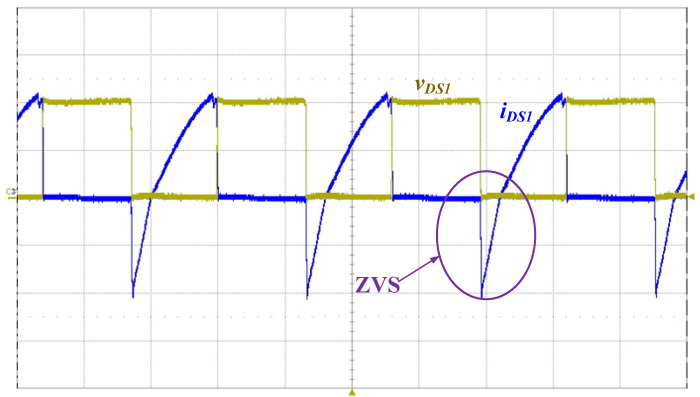
Measured waveforms of the switch voltage *v_DS_*_1_ (200 V/div) and the switch current *i_DS_*_1_ (2 A/div); time scale: 10 μs/div.

**Figure 14:**
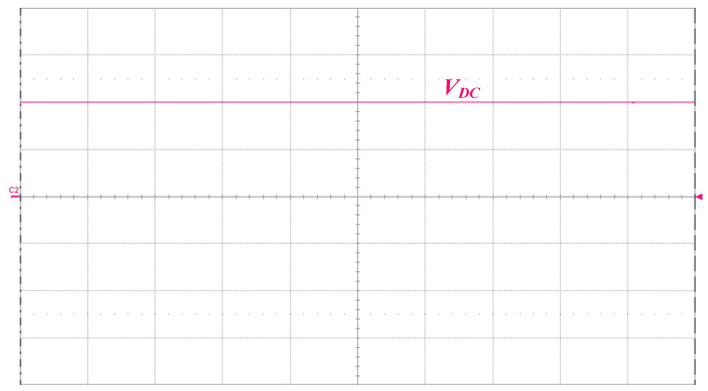
Measured waveforms of the DC-bus voltage *V_DC_* (200 V/div); time scale: 500 ns/div.

**Figure 15:**
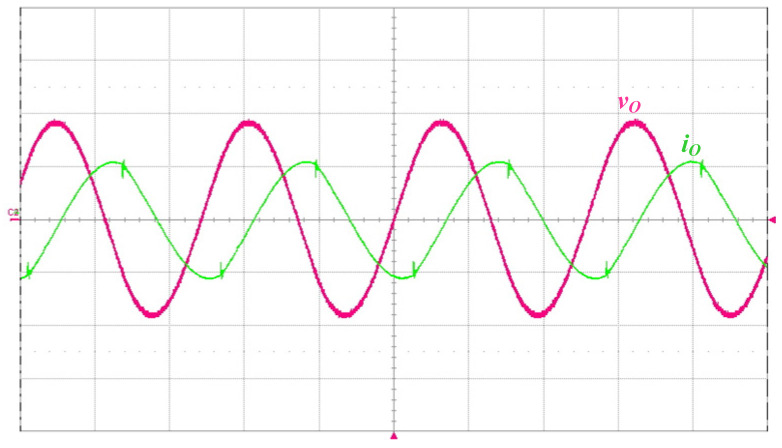
Measured waveforms of the output voltage *v_O_* (500 V/div) and the output current *i_O_* (1 A/div); time scale: 10 μs/div.

Figure 13 shows the measured waveforms of the switch voltage *v_DS_*_1_ and the switch current *i_DS_*_1_. As can be seen from the waveform diagram, ZVS occurs on the power switch in order to reduce switching losses. Figure 14 shows the measured waveform of the DC-bus voltage *V_DC_*, and the mean value of *V_DC_* is 399.6 V. Figure 15 presents the measured waveforms of the output voltage *v_O_* and the output current *i_O_*, and the output voltage *v_O_* leads the output current *i_O_*, as can be seen from the figure.

**Figure 16:**
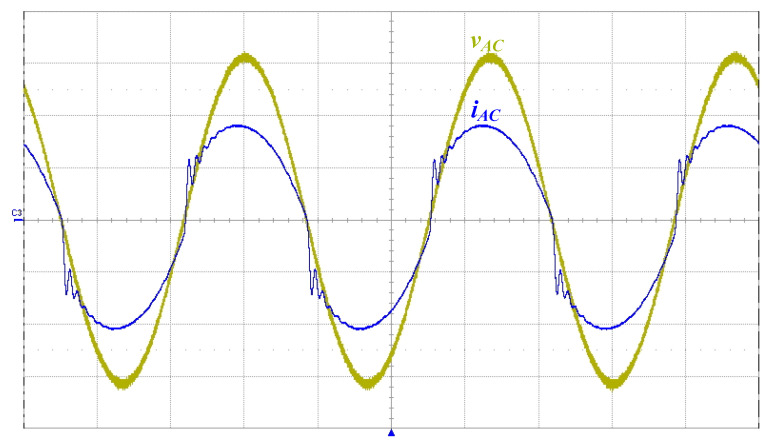
Measured waveforms of the input utility-line voltage *v_AC_* (50 V/div) and the input current *i_AC_* (0.5 A/div); time scale: 5 ms/div.

**Figure 17:**
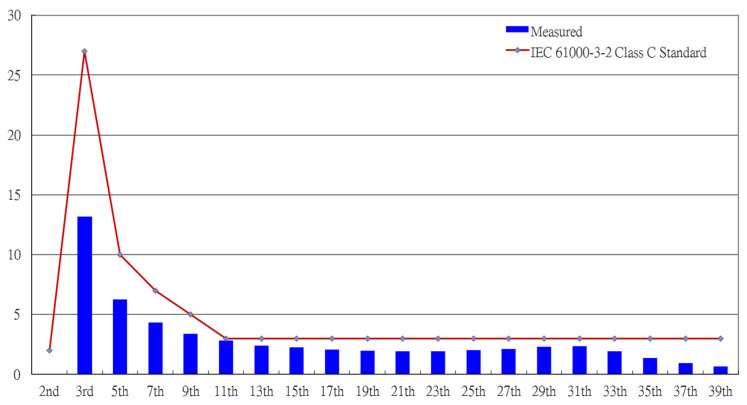
Measured harmonics of the input utility-line current in comparison with the IEC 61000-3-2 class C standard.

Figure 16 shows the measured input utility-line voltage *v_AC_* and current *i_AC_*. As can be seen from the waveform diagram, the ICS function was realized in the proposed driver circuit. Figure 17 shows the harmonic components of the AC input current measured with a power analyzer (Tektronix PA 4000) and compared to the IEC 61000-3-2 Class C standard [28]. As can be seen from the figure, all current harmonics met the requirements.

**Figure 18:**
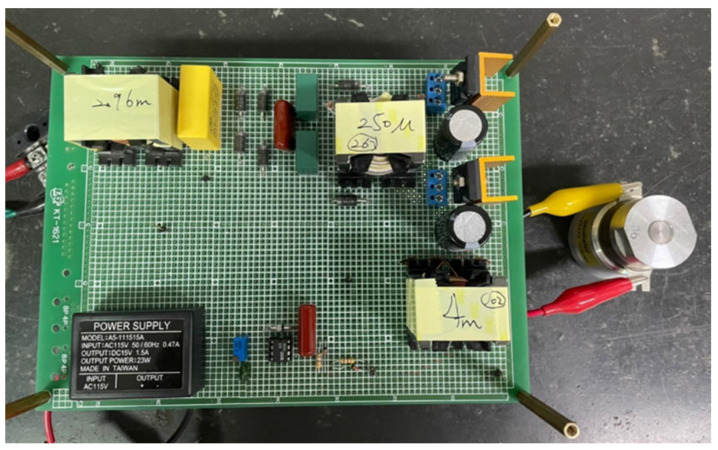
The photograph of the prototype driver circuit proposed in this paper for a piezoelectric ceramic actuator.

Furthermore, the measured total-harmonic distortion (THD) of the input utility-line current and the power factor in the proposed driver circuit were 15.432% and 0.9729, respectively. Additionally, a photograph of the prototype drive circuit for powering the piezoelectric ceramic actuator developed in this paper is shown in Figure 18.

## 4. Conclusions

This paper introduces the principle of piezoelectricity, the application of piezoelectric ceramic actuators, and the existing three-stage and two-stage piezoelectric ceramic actuator drive circuit architectures, and proposes a new two-stage drive circuit architecture for piezoelectric ceramic actuators. The drive circuit developed in this paper consists of a full-wave bridge rectifier in the front stage and a stacked boost DC-to-DC converter integrating with a half-bridge series resonant DC to AC converter as the rear stage to form a new two-stage circuit architecture to provide energy to the piezoelectric ceramic actuator with power factor correction. Experimental results obtained from a 50 W-rated prototype drive circuit at a 110 V input utility-line voltage have sufficiently demonstrated a high power factor (>0.97) and low total harmonic distortion (<16%) of the input utility-line current, and two power switches are provided with the ZVS feature in the proposed driver circuit.

## Figures and Tables

**Table 1:** Specifications of the experimental piezoelectric ceramic actuator.

Parameter	Value
Rated Power *P_O_*	50 W
Resonant Resistance *R_m_*	25 Ω
Static Capacitance *C_p_*	4000 pF
Resonant Frequency *f_r_*	40 ± 0.5 kHz
Outer Diameter of Radiating Surface	45 mm
Outer Diameter of the Ceramic Ring	35 mm
Height	54 mm
Weight	244 g

**Table 2:** The components used in the prototype driver circuit for supplying a piezoelectric ceramic actuator.

Parameter/Component	Value
Diode *D*_1_, *D*_2_, *D*_3_, *D*_4_	MUR460
Filter Inductor *L_f_*	2.96 mH
Filter Capacitor *C_f_*	1 μF
Capacitor *C_rec_*; *C_in_*_1_, *C_in_*_2_	1 μF; 0.33 μF
Diode *D_B_*_1_, *D_B_*_2_	MUR460
DC-linked Capacitor *C_DC_*_1_, *C_DC_*_2_	150 μF (Two capacitors in series connection)
Power Switches *S*_1_, *S*_2_	35N60CFD
Resonant Inductor *L_r_*	4 mH
Series Inductor *L_B_*	250 μH

## Data Availability

Not applicable.

## References

[1] Piezoelectricity. https://en.wikipedia.org/wiki/Piezoelectricity.

[2] Prathamesh N., Leonard B., Bond J. (2018). Resonance analysis of a high temperature piezoelectric disc for sensitivity characterization. Ultrasonics.

[3] Agbossou K., Dion J.L., Carignan S., Abdelkrim M., Cheriti A. (2000). Class D Amplifier for a Power Piezoelectric Load. IEEE Trans. Ultrason. Ferroelectr. Freq. Control.

[4] Honarvar F., Salehi F., Safavi V., Mokhtari A., Sinclair A.N. (2013). Ultrasonic monitoring of erosion/corrosion thinning rates in industrial piping systems. Ultrasonics.

[5] Kauczor C., Frohleke N. Inverter Topologies for Ultrasonic Piezoelectric Transducers with High Mechanical Q-Factor. Proceedings of the Annual IEEE Power Electronics Specialists Conference.

[6] Xu J., Lin S.Y., Hu J. (2019). Electromechanical equivalent circuit and coupled vibration of the radially composite cylindrical piezoelectric transducer. Sens. Actuators A Phys..

[7] Tsujino J., Hongoh M., Yoshikuni M., Miura H., Ueoka T. Welding Characteristics and Temperature Rises of Various Frequency Ultrasonic Plastic Welding. Proceedings of the IEEE Ultrasonics Symposium.

[8] Guo S.F., Chen S.T., Zhang L., Liew W.H., Yao K. (2019). Direct-write piezoelectric ultrasonic transducers for pipe structural health monitoring. NDT E Int..

[9] Yu T., Lee H., Lee D., Song S., Kim D., Park S. Design of LC Resonant Inverter for Ultrasonic Metal Welding System. Proceedings of the 2008 International Conference on Smart Manufacturing Application.

[10] Wang J.J., Li W.J., Lan C.M., Wei P.J., Luo W. (2020). Electromechanical impedance instrumented piezoelectric ring for pipe corrosion and bearing wear monitoring: A proof-of-concept study. Sens. Actuators A Phys..

[11] Volosencu C. Control System for Ultrasonic Welding Devices. Proceedings of the IEEE International Conference on Automation, Quality and Testing.

[12] Lin S. (2008). The radial composite piezoelectric ceramic transducer. Sens. Actuators A Phys..

[13] Lee J.H., Lee H.C., Choi J.H., Park S.J., Nam H.G. 10kW Industrial Ultrasonic Welder Design. Proceedings of the 31st International Telecommunications Energy Conference.

[14] Cheng C., Wang S., Tian H., Lin S. (2021). Study on the bending vibration of bimorph rectangular transducer based on type 2-2 piezoelectric composites. Ultrasonics.

[15] Ma K.H., Chang W.C., Lee Y.C. A New Tracking Method with FPGA Chip for Ultrasonic Welding System. Proceedings of the 2009 International Conference on Power Electronics and Drive Systems.

[16] (2017). Piezoelectric Ceramics, TOKIN. https://www.tokin.com/english/product/pdf_dl/piezoelectricceramics.pdf.

[17] Zhou Y., Zhou B., Li S. Driving Performance Analysis of a Novel Piezoelectric Actuator. Proceedings of the 2010 International Conference on Measuring Technology and Mechatronics Automation.

[18] Jabbar H., Jung H.J., Cho J.Y., Sung T.H. (2016). Non-resonant piezoelectric transformer based power converter for ultra-low-power electronic devices. Sens. Actuators A Phys..

[19] Ghasemi N., Zare F., Davari P., Weber P., Langton C., Ghosh A. Power electronic converters for high power ultrasound transducers. Proceedings of the 2012 7th IEEE Conference on Industrial Electronics and Applications (ICIEA).

[20] Wang S., Shan J.J., Lin S.Y. (2022). Radial vibration analysis for functionally graded ring piezoelectric transducers based on electromechanical equivalent circuit method. Ultrasonics.

[21] Lian Y., Gao C., Liu X. A hybrid driving strategy for piezoelectric actuator. Proceedings of the 2013 International Conference on Mechatronic Sciences, Electric Engineering and Computer (MEC).

[22] Cheng L., Kang Y., Chen C. (2014). A Resonance-Frequency-Tracing Method for a Current-Fed Piezoelectric Transducer. IEEE Transactions on Industrial Electronics.

[23] Lai J.A. (2004). Two-Stage Driver for Piezoelectric Actuator. Master’s Thesis.

[24] Jittakort J., Sangswang A., Naetiladdanon S., Chudjuarjeen S., Koompai C. LCCL Series Resonant Inverter for Ultrasonic Dispersion System with Resonant Frequency Tracking and Asymmetrical Voltage Cancellation Control. Proceedings of the IECON2015.

[25] Jittakort J., Sangswang A., Naetiladdanon S., Koompai C., Chudjuarjeen S. (2017). Full Bridge Resonant Inverter Using Asymmetrical Control with Resonant-frequency Tracking for Ultrasonic Cleaning Applications. J. Power Electron..

[26] Cheng C.A., Cheng H.L., Chang C.H., Chang E.C., Lan L.F., Hsu H.F. A Novel Driver Circuit for Piezoelectric Ceramic Actuator Featuring with Input-Current-Shaping and Soft-Switching. Proceedings of the 25th International Conference on Mechatronics Technology (ICMT 2022).

[27] Cheng C.A., Cheng H.L., Chang C.H., Chang E.C., Tsai C.Y., Lan L.F. (2021). A Novel and Cost-Effective Drive Circuit for Supplying a Piezoelectric Ceramic Actuator with Power-Factor-Correction and Soft-Switching Features. Micromachines.

[28] Wikipedia, the Free Encyclopedia. “IEC 61000-3-2”. https://en.wikipedia.org/wiki/IEC_61000-3-2.

